# Dual actionability of BMI1 activation and mitotic vulnerability defines adaptive Osimertinib resistance in EGFR-mutant NSCLC

**DOI:** 10.1038/s41419-026-08952-2

**Published:** 2026-06-10

**Authors:** Paolo Armanetti, Eva Cabrera San Millan, Maddalena Di Nardo, Raffaella Mercatelli, Chiara Cavallini, Stefania Crucitta, Marzia Del Re, Alice Chiodi, Ettore Mosca, Arianna Consiglio, Nicola Losito, Antonio Musio, Luca Menichetti, Emilia Bramanti, Azhar Ali, Elena Levantini, Giorgia Maroni

**Affiliations:** 1 CNR-Consiglio Nazionale delle Ricerche, Istituto di Fisiologia Clinica (IFC-CNR), Area della Ricerca di Pisa, Pisa, Italy; 2 CNR-Consiglio Nazionale delle Ricerche, Istituto di Tecnologie Biomediche (ITB-CNR), Area della Ricerca di Pisa, Pisa, Italy; 3 Chemical-Clinical Analyses Unit, ASST Grande Ospedale Metropolitano Niguarda, Milano, Italy; 4 Saint Camillus International University of Medical and Health Sciences, Rome, Italy; 5 CNR-Consiglio Nazionale delle Ricerche, Istituto di Tecnologie Biomediche (ITB-CNR), Segrate (Milano), Segrate, Italy; 6 CNR-Consiglio Nazionale delle Ricerche, Istituto di Tecnologie Biomediche (ITB-CNR), Bari, Italy; 7 CNR-Consiglio Nazionale delle Ricerche, Istituto di Chimica dei Composti OrganoMetallici (ICCOM-CNR), Area della Ricerca di Pisa, Pisa, Italy; 8 Cancer Science Institute of Singapore, National University of, Singapore, Singapore; 9 Fondazione Pisana per la Scienza ONLUS (FPS), San Giuliano Terme, Pisa, Italy; 10 Present Address: CNR-Consiglio Nazionale delle Ricerche, Istituto Nanoscienze –NEST-SNS, Pisa, Italy; 11 Present Address: CNR-Consiglio Nazionale delle Ricerche, Istituto di Ricerca sulle Acque (IRSA), Bari, Italy

**Keywords:** Non-small-cell lung cancer, Cancer therapeutic resistance, Mitotic spindle, Cell invasion

## Abstract

Non-small cell lung cancer (NSCLC) remains a leading cause of cancer mortality worldwide. Tumors carrying activating epidermal growth factor receptor (EGFR) mutations initially respond to tyrosine kinase inhibitors (TKIs), with Osimertinib representing the current standard of care. However, acquired resistance inevitably develops, involving both genetic and non-genetic mechanisms, the latter playing a major role in sustaining cellular plasticity and promoting tumor aggressiveness. Here, we show that Osimertinib-resistant H1975 cells acquire a more aggressive phenotype than their parental (Par) counterparts, characterized by enhanced migratory behavior and transcriptional enrichment of BMI1 target genes, as well as mitotic defects and concomitant alterations in expression of mitotic cell-cycle pathways. Despite unchanged proliferation, resistant cells display increased mitotic activity and frequent cytokinetic defects, revealing a dependency on mitotic machinery and microtubule integrity, an Achilles’ heel created by adaptive resistance. Functionally, BMI1 overexpression in Par cells recapitulates both resistance and enhanced migration, highlighting its central role in driving the resistant phenotype. Exploiting these vulnerabilities, Unesbulin (PTC596), a tubulin-binding agent with BMI1 inhibitory activity, triggers mitotic catastrophe, mechanistically induces apoptosis in vitro and drives regression of resistant xenografts in vivo. Our findings establish BMI1 as a key mediator of Osimertinib resistance and aggressiveness, uncovering a mutation-context-dependent mitotic vulnerability that can be therapeutically exploited, providing a rationale for targeting BMI1 and mitotic abnormalities to overcome resistance in T790M/L858R backgrounds.

## Introduction

Non-small cell lung cancer (NSCLC) accounts for ~85% of lung cancer cases worldwide and remains a leading cause of cancer-related mortality [1]. Tumors harboring activating mutations in the epidermal growth factor receptor (EGFR) represent a clinically relevant subgroup, characterized by high sensitivity to EGFR tyrosine kinase inhibitors (TKIs) [2]. Third-generation TKIs, such as Osimertinib, initially developed to overcome T790M-mediated resistance, are now adopted as first-line settings due to their ability to target both sensitizing and resistant EGFR mutations [2].

Despite initial responses, the development of acquired resistance to Osimertinib is inevitable, arising typically within 1-2 years of treatment initiation [3]. We previously reported a mitochondria-centered adaptive program, in which mitochondrial remodeling and activation of the pyruvate-acetaldehyde-acetate (PAA) pathway converge to promote cell survival and drug tolerance in Osimertinib-resistant NSCLC [4, 5]. Several other mechanisms have been described, including EGFR secondary mutations (e.g., C797S), activation of bypass signaling pathways, histological transformation, and engagement of epithelial-to-mesenchymal transition (EMT) programs [3]. Non-genetic adaptive mechanisms are increasingly recognized as key contributors, supporting cellular plasticity, and survival under therapeutic pressure [6]. However, the functional consequences of these adaptations, particularly in relation to tumor aggressiveness and invasive potential, remain incompletely understood.

Polycomb group proteins, particularly BMI1, a core component of the Polycomb Repressive Complex 1 (PRC1), is a key regulator of stemness, EMT, DNA repair, and therapy tolerance in multiple cancers [7–9]. In NSCLC, elevated BMI1 expression has been associated with poor prognosis, enhanced metastatic potential, and resistance to chemo- and radio-therapy [2, 10–14]. Nevertheless, its role in Osimertinib-resistant EGFR-mutant (EGFRm) NSCLC remains poorly characterized.

Here, we investigated Osimertinib-resistant H1975 cells (H1975 Osi-R), revealing enrichment of BMI1 target genes and mitotic pathways, including spindle assembly, G2/M checkpoint, as well as Myc and E2F transcriptional programs, and DNA repair processes. Overall, resistant cells display a transcriptional profile that supports survival and mitotic adaptation. Functionally, Osi-R cells displayed enhanced migration and frequent mitotic abnormalities, showing they acquired dependency on mitotic regulation and cell-cycle, as well as microtubule dynamics, potentially predisposing them to mitotic stress and genomic instability.

Unesbulin (PTC596), a tubulin-targeting agent that also reduces BMI1 levels, effectively exploited this dual vulnerability, inducing apoptosis and tumor regression.

Unesbulin has been evaluated in a phase I clinical trial and was well tolerated in patients with advanced solid tumors [15]. Preclinical studies indicate that Unesbulin destabilizes microtubules, impairs mitotic progression, and triggers apoptotic cell death [15–17]. A phase II/III trial has been registered to assess its safety and efficacy (EudraCT 2022-000073-12).

By testing the efficacy of Unesbulin both in vitro and in vivo, we demonstrate that BMI1 acts as a key mediator of Osimertinib resistance and aggressiveness, while mitotic stress represents an actionable weakness in Osimertinib-resistant EGFRm NSCLC. Overall, our findings provide a strong rationale for therapeutic targeting of BMI1 and mitotic vulnerabilities in EGFRm NSCLC that acquired resistance to Osimertinib.

## Results

### H1975 Osi-R cells acquire enhanced migration and mitotic dependency

To investigate the mechanisms underlying Osimertinib resistance, we generated a resistant H1975 cell line [4, 5]. IC50 assays (Supplementary Fig. 1A) confirmed the resistant phenotype: after 72 h of treatment with 1 μM Osimertinib, Osi-R cells retained 80% viability, while parental cells dropped to 40% (*p* = 0.0005).

**Fig. 1:**
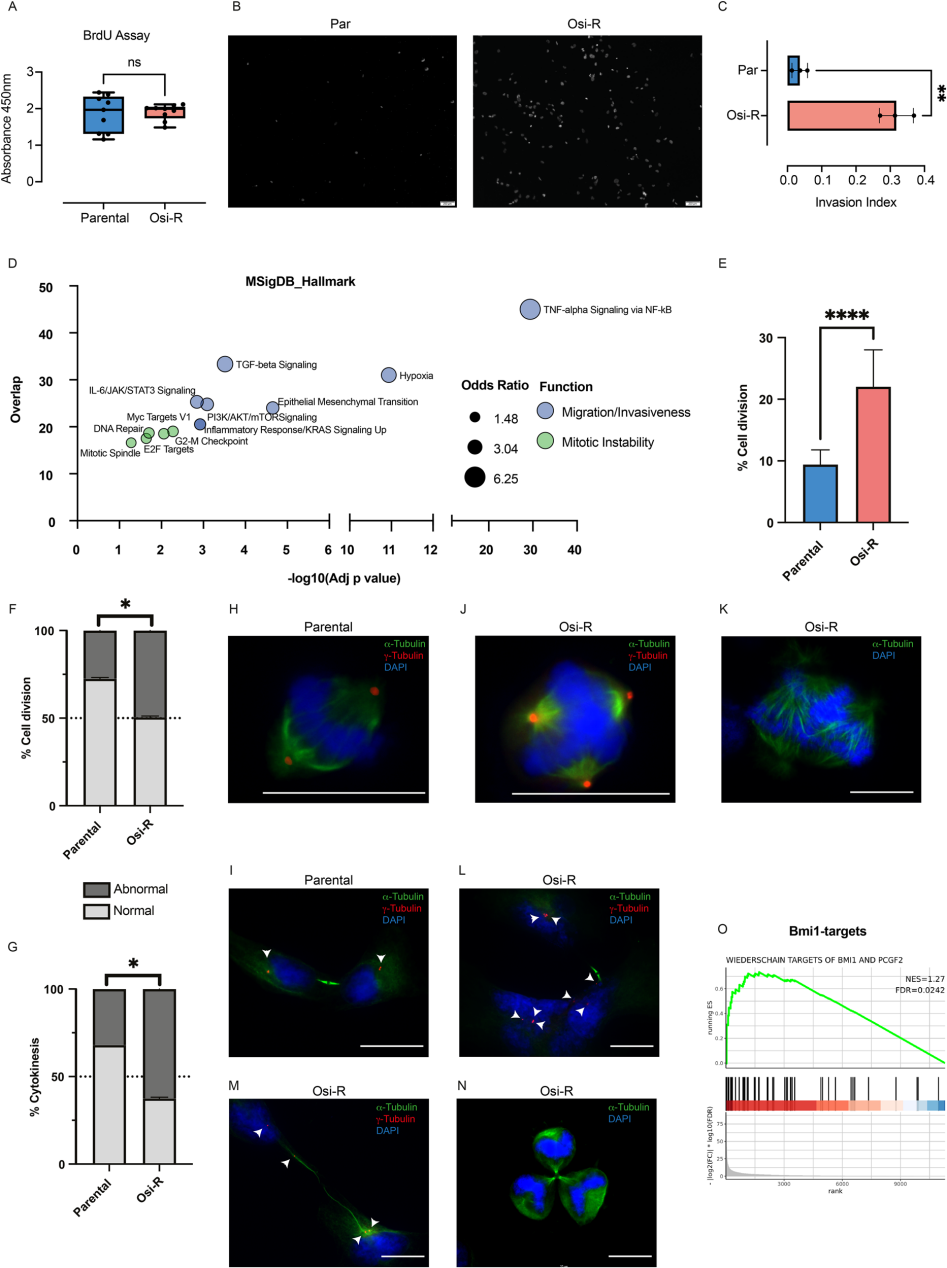
Migration assays and transcriptomic analysis reveal enhanced invasiveness and mitotic dependency in Osimertinib-resistant EGFRm NSCLC cells. **A** BrdU cell proliferation assays in H1975 Par (blue) and Osi-R (red) cells. Error bars represent Standard Deviation (SD); no significant differences in proliferation were detected (ns, not significant, two-tailed Welch’s T test, *n* = 9). **B** Representative transwell migration images with DAPI-stained nuclei in Par (left panel) and Osi-R H1975 cells (right panel). **C** Quantification of transwell migration. Error bars represent SD; *p*-value is indicated (**p < 0.0021, Welch’s T test, *n* = 3). **D** RNA-seq pathway enrichment (EnrichR, MSigDB_Hallmark) in Osi-R cells showing statistically significant pathways associated with migration/invasiveness (blue) and mitotic instability (green). Dot size indicates odds ratio. **E** Percentage of dividing cells in Par (blue) and Osi-R (red) populations (****p < 0.0001, unpaired t-test, two-tailed with Welch’s correction, *n* = 7). Percentage of normal (light grey) vs. abnormal (dark grey) mitotic divisions **F** and cytokinesis **G** in Par and Osi-R (*p < 0.05, Fisher´s exact test). Representative fluorescence microscopy images (100X) of cell division, stained for α-Tubulin (green), γ-Tubulin (red), and DAPI (blue). Bar indicates 20 μm. Panels **H, I**: normal metaphase **H** and cytokinesis **I** in Par cells. Panels **J–N**, abnormal metaphase **J, K** and cytokinesis **L, N** in Osi-R cells. White arrows indicate centrioles marked by γ-Tubulin. **O** Gene set enrichment analysis (GSEA) of BMI1 target genes showing positive enrichment in Osi-R cells. Normalized Enrichment Score (NES) = 1.27; FDR: 0.0242.

Since we previously reported that H1975 Osi-R cells undergo metabolic rewiring [4, 5]. We first assessed whether these alterations impacted proliferation. BrdU incorporation assays revealed no significant differences between Osi-R and Par cells after 24 h (Fig. 1A), indicating that resistance does not involve increased proliferative ability.

Despite comparable proliferation rates, Osi-R cells displayed a markedly more aggressive phenotype. Transwell migration assays revealed enhanced migratory capacity (Fig. 1B), as shown by DAPI staining, with a higher number of migrated Osi-R cells compared to Par cells (Fig. 1B). Quantification confirmed an ~8.8-fold increase in the migration index (Osi-R = 0.3179, Par=0.036, *p* = 0.0038) (Fig. 1C). Consistently, wound-healing assays further confirmed their heightened invasiveness in that H1975 Osi-R cells demonstrated faster gap closure, starting repopulating the scratched area as early as 8 h and achieved complete closure within 14 h (Supplementary Fig. 1B, C). These results demonstrate that Osimertinib resistance is associated to increased cell motility and invasive potential.

To define the molecular programs driving this phenotype, we performed transcriptomic analysis by RNA-seq. Pathway enrichment analysis (EnrichR, MSigDB_Hallmark database) of our previously reported dataset^4^ revealed that Osi-R cells display enrichment of multiple pathways linked to invasiveness, including TNF-alpha Signaling via NF-kB, Hypoxia, TGF-beta Signaling, Epithelial Mesenchymal Transition, PI3K/AKT/mTOR Signaling, Inflammatory Response, KRAS Signaling Up, and IL-6/JAK/STAT3 Signaling (Fig. 1D). These transcriptional programs are consistent with the enhanced migratory and stress-adaptive behavior observed functionally.

In parallel, Osi-R cells also displayed enrichment of pathways/gene signatures related to mitotic regulation, including mitotic spindle assembly, G2/M checkpoint, E2F and Myc targets, and DNA repair (Fig. 1D). Although early cell-cycle phases remain largely intact, given that DNA synthesis and S-phase progression were not significantly altered as demonstrated by BrdU incorporation assay, RNA-seq data indicate defects emerging specifically during mitosis. This transcriptional profile suggests enhanced mitotic activity and potential mitotic instability in resistant cells.

Consistently, immunofluorescence (IF) analyses of α/γ-tubulin and DAPI confirmed a higher mitotic index in Osi-R cell compared with Par controls. Quantitative analysis revealed that 22% of Osi-R cells were dividing versus 9% of Parental cells (*p* < 0.0001), indicating elevated mitotic activity in resistant cells (Fig. 1E). However, Osi-R cells also exhibited a significantly higher frequency of abnormal mitosis (49% vs. 27% in Par cells, *p* = 0.05) (Fig. 1F) and cytokinesis defects (65% vs. 32%, *p* = 0.0241) (Fig. 1G).

IF images further illustrate these abnormalities. Majority of Par cells showed well-organized mitotic structures, including normal metaphase spindles with properly positioned centrioles (Fig. 1H) and symmetric cytokinesis with regular morphology (Fig. 1I). On the contrary, Osi-R cells displayed a broad spectrum of mitotic abnormalities during metaphase, including disorganized or fragmented microtubule arrays, (Fig. 1J) multipolar spindles (Fig. 1K), supernumerary (often more than two per cell) (Fig. 1L) or mislocalized centrioles relative to the spindle poles (Fig. 1M). Moreover, resistant cells frequently exhibited elongated (Fig. 1M) or irregular cytokinetic bridges (Fig. 1N), often containing retained or supernumerary centrioles (Fig. 1 L, M), defects that collectively are observed in Par cells at much lower extents. These features reflect a major deregulation of mitotic control and spindle integrity in resistant cells.

Taken together, these results indicate that Osimertinib resistance in H1975 cells is not characterized by increased proliferation, but by enhanced migratory capacity and mitotic deregulation.

Gene set enrichment analysis (GSEA), performed on RNAseq data, further revealed a positive enrichment of BMI1 target genes in Osi-R cells (NES = 1.27; FDR = 0.0242) (Fig. 1O). This observation is consistent with the established role of BMI1 in supporting tumor aggressiveness sustaining stemness, chromatin remodeling, cell cycle progression, and mitotic control. While this transcriptional reprogramming may enhance tumor adaptability and resilience under therapeutic pressure, the concomitant accumulation of mitotic and cytokinetic defects exposes a potential vulnerability that may be therapeutically exploited.

### Pharmacological targeting via Unesbulin induces apoptosis of H1975 Osi-R cells in vitro

Given the reprogramming of Osi-R cells, which includes dependency on BMI1 and deregulated mitotic processes, we next investigated whether this phenotype could reveal a pharmacologically targetable vulnerability. We reasoned that the dual activity of Unesbulin, a tubulin-binding agent with BMI1 inhibitory activity, might effectively exploit the dual weakness displayed by Osi-R cells, by simultaneously disrupting BMI1-driven transcriptional programs and concomitantly affecting microtubule integrity.

**Fig. 2:**
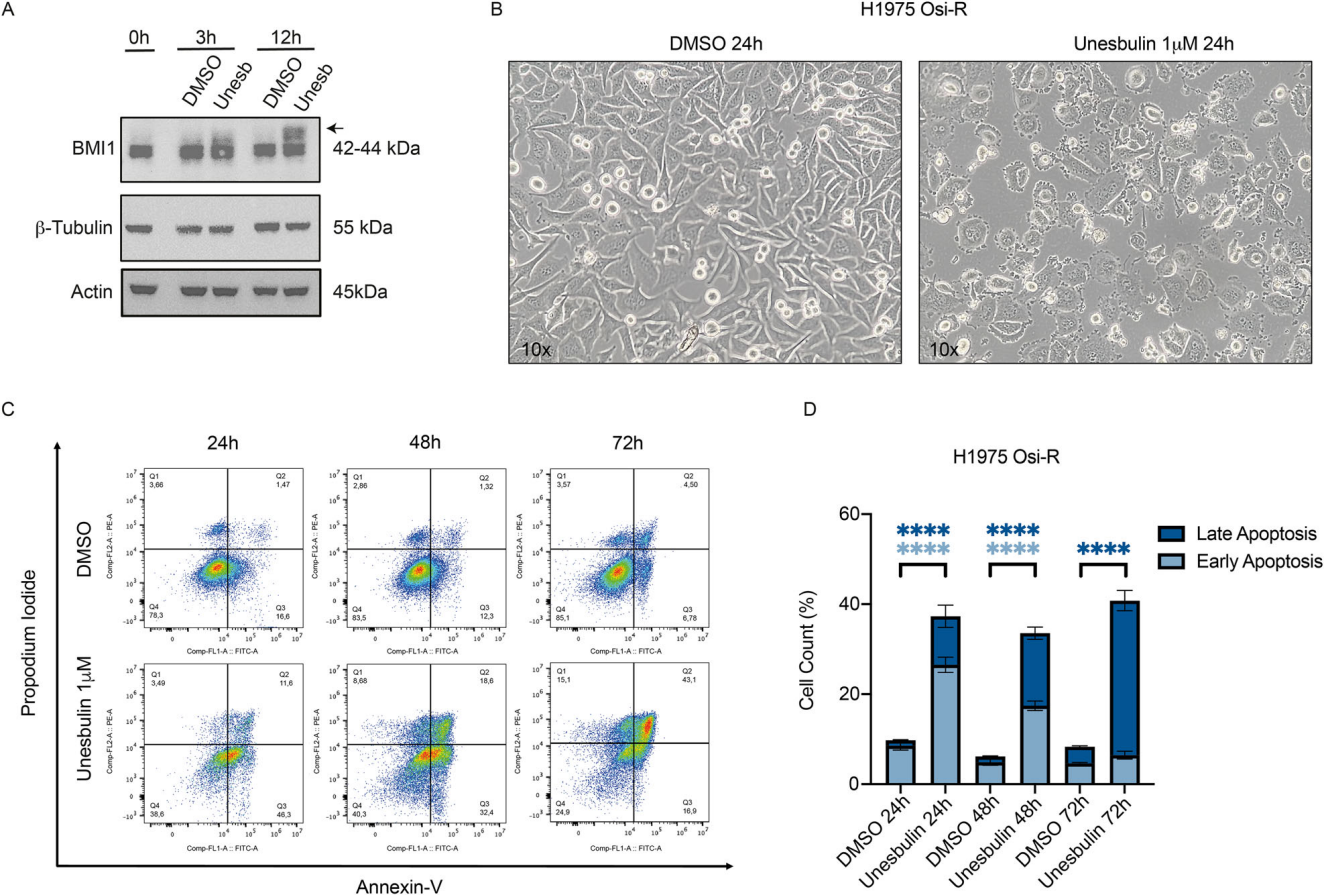
Unesbulin treatment induces apoptosis in Osimertinib-resistant EGFRm NSCLC cells. **A** Western Blot analyses of H1975 Osi-R after 0 h, 3 h and 12 h of DMSO and Unesbulin 1 μM treatment. Protein lysates were immunoblotted with an antibody against BMI1 and β-Tubulin. β-Actin was used as a loading control. Expected molecular weights (kDa) are indicated. BMI1 hyperphosphorylated band is indicated by the arrow. **B** Representative image of H1975 Osi-R cells treated with DMSO (control, left panel) or Unesbulin (right panel) after 24 h. Images were acquired at 10× magnification. **C** Annexin V/PI flow cytometry analysis of H1975 Osi-R cells treated with DMSO or Unesbulin for 24, 48, and 72 h. Plots show progressive increases in Annexin V+ (FITC-A channel) (early apoptotic) and Annexin V + /PI+ (PE-A channel) (late apoptotic) populations in Unesbulin-treated cells. **D** Percentage of apoptotic populations in DMSO- versus Unesbulin-treated cells. Early apoptosis is shown in light blue, and late apoptosis in dark blue. Error bars represent SD; *p*-values are indicated (****p < 0.0001, 2-way ANOVA, *n* = 3).

To test this hypothesis, we evaluated the response of Osi-R cells to Unesbulin (1 μM). In line with the known Unesbulin mechanism of action, Western Blot analysis showed progressive BMI1 hyperphosphorylation after 3 h and 12 h of treatment, accompanied by 35% β-Tubulin decrease (Fig. 2A), confirming affected BMI1 signaling and microtubule stability.

Remarkably, a strong cytotoxic response was already present 24 h post-treatment, with the majority of Osi-R cells displaying extensive cell death (Fig. 2B). Flow cytometry analysis adopting Annexin-V/Propidium Iodide (PI) staining confirmed apoptosis induction. Specifically, after 24 h of Unesbulin treatment, Osi-R cells showed a significant 3.1-fold increase in Annexin-V^+^ cells as compared to DMSO-treated controls (26.53% ± 1.68 Vs. 8.56 ± 0.97% *p* = 8.3×10^-14^) (Fig. 2C, D), indicating early apoptotic activation. At later time points, a progressive rise in double positive (Annexin-V^+^ / PI^+^) cells was observed upon treatment (16.13 ± 1.36% and 34.33 ± 2.26%, respectively, at 48 h and 72 h), as compared to DMSO (1.25 ± 0.13% and 3.67 ± 0.20%) (Fig. 2C, D), consistent with a time-dependent increase in late-stage apoptosis relative to controls (*p* = 4×10^-^^12^ and 2×10^-^^14^, respectively, Fig. 2C, D).

Collectively, these in vitro data demonstrate that Osi-R cells are highly sensitive to Unesbulin treatment. The data support a model in which BMI1 inhibition and microtubule destabilization act in concert to trigger catastrophic mitotic failure and apoptosis in Osi-R cells, thereby converting their acquired dependencies into therapeutic vulnerabilities.

### Unesbulin induces regression of H1975 Osi-R xenografts in vivo

**Fig. 3:**
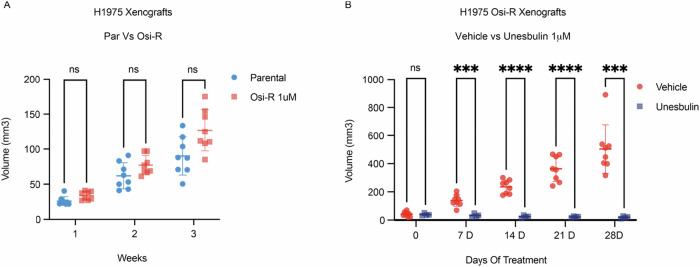
Unesbulin treatment induces tumor regression in Osimertinib-resistant EGFRm NSCLC cells in vivo. **A** Tumor growth kinetics of H1975 Par (blue, *n* = 8) and Osi-R (red, *n* = 8) xenografts. Error bars represent SD; no significant differences were detected (ns, not significant). **B** Tumor growth kinetics of H1975 Osi-R treated with Vehicle (red, *n* = 8) or Unesbulin (blue, *n* = 4). Error bars represent SD; p-values are indicated (ns, not significant, ***p < 0.0002, ****p < 0.0001, multiple unpaired T tests with Welch’s correction, Holm-Šídák method).

First, to test the proliferation ability of Osi-R cells, we evaluated the in vivo growth capacity of xenografts derived from H1975 resistant compared to their parental counterparts. Both Osi-R and Par cells were transplanted subcutaneously into immunocompromised mice, and tumor growth (mm³) was monitored weekly by ultrasound (US), starting 10 days post-implantation. Consistent with in vitro proliferation assays, Osi-R and Par xenografts displayed comparable growth rates over a 3-week period (Fig. 3A), confirming that resistance is not associated with increased baseline proliferation.

As a second step, encouraged by the in vitro efficacy of Unesbulin, we next evaluated its in vivo anti-tumor activity by testing H1975 Osi-R-derived xenografts. Once Osi-R tumors reached ~40 mm³, mice received biweekly Unesbulin treatment (12 mg/Kg). Notably, while vehicle-treated tumors continued to grow to an average final volume of ~500 mm³, Unesbulin-treated tumors regressed to ~20 mm³, representing a 25-fold reduction in tumor volume (*p* = 3.5×10^-5^) (Fig. 3B). These data demonstrate that Unesbulin efficiently targets the vulnerabilities of Osi-R tumors in vivo, thus translating the mitotic and BMI1-dependent sensitivity observed in vitro, into relevant anti-tumor activity.

### Bmi1 upregulation in H1975 parental cells resembles both the in vitro and in vivo Osi-R phenotype

**Fig. 4:**
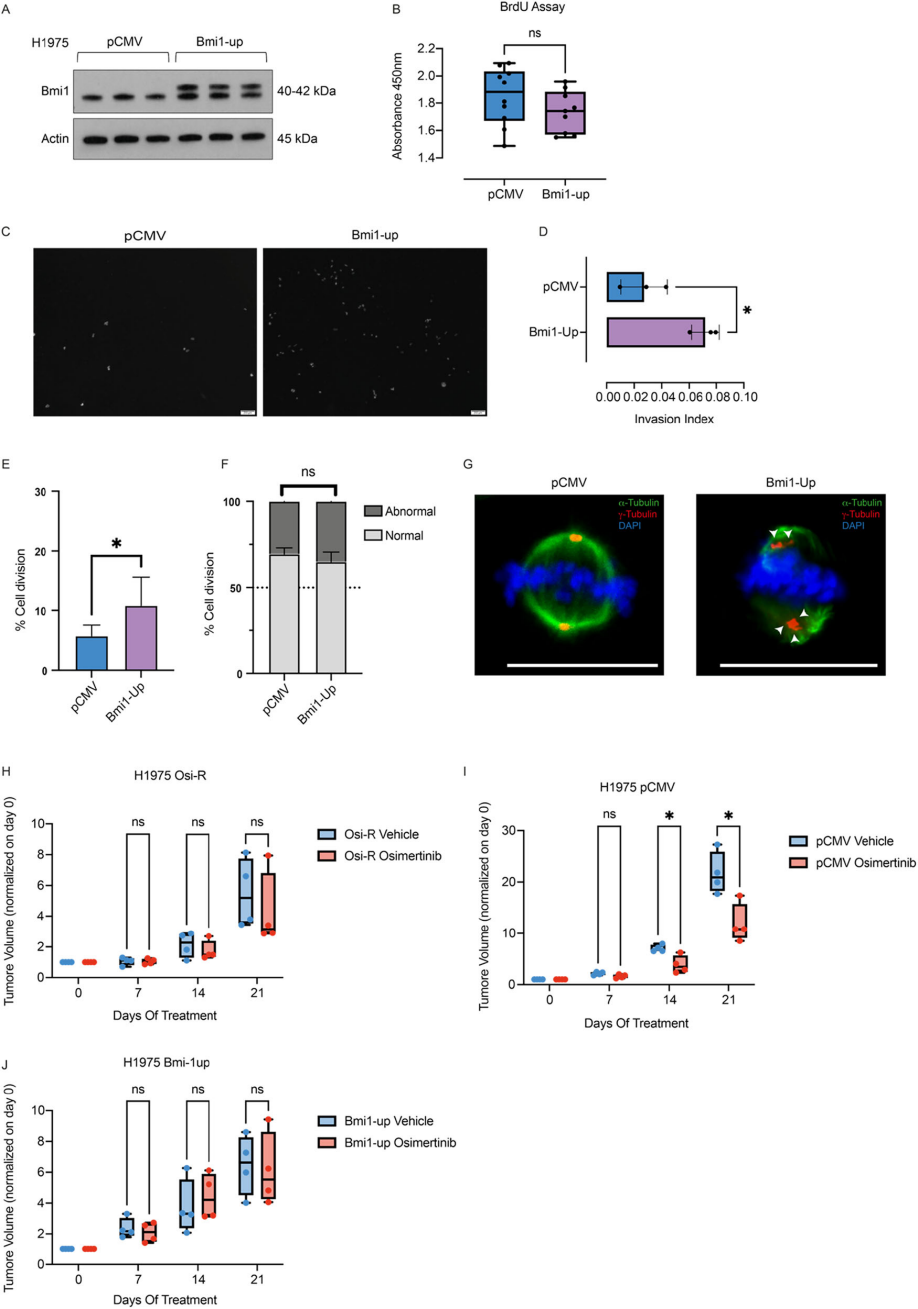
BMI1 overexpression enhances migration and confers resistance to Osimertinib in EGFRm NSCLC cells. **A** Western Blot analyses of H1975 cells transfected with pCMV3 (pCMV cells) and pCMV3-Bmi1-up (Bmi1-up cells). Protein lysates were immunoblotted with an antibody against BMI1. The higher FLAG-tagged band is indicated by the arrow. β-Actin was used as loading control. Expected molecular weights (kDa) are indicated. **B** BrdU cell proliferation assays in H1975 pCMV3 (blue) and pCMV3-Bmi1-up (purple). Error bars represent SD; no significant differences in proliferation were detected (ns, not significant, unpaired two-tailed Welch’s T test, *n* = 10). **C** Transwell migration images of DAPI-stained nuclei in H1975 pCMV cells (left panel) and Bmi1-up cells (right panel). **D** Quantitative analysis of the transwell migration assay; pCMV cells are indicated in blue and Bmi1-up cells in purple. Error bars represent SD; p-value is indicated (*p < 0.0332, unpaired two-tailed Welch’s T test, *n* = 3). **E** Percentage of dividing cells in pCMV (blue) and Bmi-up (purple) populations; Bmi-up cells show increased mitotic figures (*p < 0.0332, unpaired T-test with Welch’s correction, *n* = 7). **F** Percentage of normal (light grey) versus abnormal (dark grey) mitotic divisions in pCMV and Bmi1-up. **G** Representative fluorescence microscopy images (100X) of cell division, stained for α-Tubulin (green), γ-Tubulin (red), and DAPI (blue). Scale bar, 20 μm. Left panel shows normal metaphase in pCMV cells; right panel show abnormal metaphase in Bmi1-up cells. White arrows indicate centrioles marked by γ-Tubulin. Tumor growth kinetics of xenografts subcutaneously injected with **H** Osi-R, **I** with pCMV, and **J** Bmi1-up cells and treated with vehicle (blue) or Osimertinib (red). Tumor volume ratios were normalized to day 0. Error bars represent SD; *p*-values are indicated (ns, not significant; *p < 0.0032, multiple unpaired T Tests with Welch’s correction, no correction for multiple comparisons, *n* = 4).

To functionally assay BMI1 direct contribution to driving the resistant phenotype, we overexpressed BMI1 in H1975 parental cells using a pCMV3-BMI1 construct (Bmi1-up), comparing them to cells transfected with the empty pCMV3 vector (control). Western Blot analysis confirmed major BMI1 overexpression in Bmi1-up cells, detected as flagged band (Fig. 4A). To further characterize the effects of Bmi1 upregulation in parental cells, we performed proliferation and migration assays. While BrdU assays revealed no significant differences in proliferation between Bmi1-up and control cells at 24 h (Fig. 4B), importantly, BMI1 overexpression significantly enhanced migratory capacity. Transwell assays showed a 2.7-fold increase in migrating Bmi1-up (0.072) cells compared to control cells (0.027) (*p* = 0.0246, Fig. 4C, D). In contrast, Bmi1 down-regulation via siRNA (Supplementary Fig. 2A) decreased migratory capacity by 50%, as assessed by transwell assay (Supplementary Fig. 2B, C). These data indicated that Bmi1-up and Osi-R cells share similar behaviors in in vitro assays, and that BMI1 is per se sufficient to drive a pro-invasive phenotype in Par cells. Furthermore, similarly to Osi-R cells, Bmi1 upregulation led to a 1.88-fold increase in the percentage of dividing cells (*p* = 0.0239. Fig. 4E), accompanied by a trend toward more frequent abnormal divisions (Fig. 4F). The most prevalent abnormalities involved defects in metaphase plate organization, with supernumerary centrioles visualized by IF, resembling those observed in Osi-R cells (Fig. 4G).

To test the impact of BMI1 overexpression in in vivo settings, we generated xenografts from H1975 Osi-R, Bmi1-up, and pCMV3 cells, and treated mice biweekly with Osimertinib (10 mg/Kg). As expected, proof-of-concept, Osi-R xenografts were resistant to Osimertinib, showing no reduction in tumor growth compared to vehicle-treated counterparts after 21 days of treatment (Fig. 4H). In contrast, xenografts derived from pCMV3-transfected cells responded to Osimertinib, reflecting the Osimertinib-sensitive status of parental control cells, with significant tumor volume reduction observed as early as two weeks post-treatment (Fig. 4I). Notably, Bmi1-up xenografts displayed resistance to Osimertinib, growing comparably to vehicle-treated tumors (Fig. 4J).

Together, these findings demonstrate that BMI1 overexpression alone is sufficient to confer Osimertinib resistance in vivo, while simultaneously enhancing migratory behavior, effectively recapitulating the key features of the Osi-R phenotype. This positions BMI1 as a central node linking therapy resistance and tumor aggressiveness, providing a functional explanation for its role in shaping the adaptive landscape of Osimertinib-resistant NSCLC.

## Discussion

Our study defines the biological and molecular landscape of NSCLC cells that acquire resistance to Osimertinib, with a particular focus on the contribution of BMI1 signaling to the resistant phenotype. We show that H1975 (EGFR T790M/L858R-mutated) Osi-R cells do not display altered proliferative rates compared with Par counterparts. Instead, they undergo a marked phenotypic shift characterized by enhanced migration, invasion, and increased reliance on mitotic machinery, traits consistent with aggressive behavior under therapeutic pressure.

Even if proliferation rates remain unchanged, the enhanced migratory phenotype and transcriptional rewiring of the resistant population suggest a selective advantage, supporting the view that therapeutic escape is not solely driven by proliferative fitness but by adaptive plasticity programs that sustain survival and dissemination despite ongoing treatment [18]. This emphasizes that early intervention strategies might need to target plasticity mechanisms, in addition to proliferation, to delay or prevent relapse.

Functionally, Osi-R cells demonstrate markedly increased migratory ability. Transcriptomic analyses reveal enrichment of pro-invasive and EMT-related networks, including TGF-β, NF-κB, IL6/JAK/STAT3, and PI3K/AKT/mTOR signaling [19, 20]. Concomitant enrichment of hypoxia- and inflammation-related pathways further highlights enhanced plasticity and stress tolerance. Overall, our data are consistent with prior reports indicating that drug resistance can also arise through non-genetic mechanisms [21]. EMT-like reprogramming has been linked to drug tolerance, collective migration in metastasis, and immune evasion, thereby providing a strong survival advantage under hostile microenvironmental conditions [22, 23].

In parallel, OsiR cells also display enrichment of mitotic spindle, G2/M checkpoint, Myc and E2F targets, as well as DNA repair signatures, indicating they acquire dependency on cell-cycle regulation and abnormal microtubule dynamics to cope with mitotic stress and protect genomic integrity. While this adaptive reprogramming confers selective advantages, it also imposes biological costs, manifested as mitotic and cytokinetic defects. From an evolutionary perspective, this shift may reflect a fitness trade-off: resistance confers enhanced migratory capacity and survival under therapeutic pressure, yet it entails chronic mitotic stress, including spindle assembly and cytokinetic defects.

These mitotic vulnerabilities expose an intrinsic weakness consistent with therapy-induced *mitotic fragility* [24], whereby reliance on mitotic integrity hypersensitizes Osi-R cells to additional perturbations of microtubule dynamics or checkpoint control. Thus, mitotic instability may represent an Achilles’ heel of the resistant phenotype, providing a rationale for therapeutic interventions that target microtubule organization or mitotic checkpoint regulators, such as Aurora kinases and PLK1.

Transcriptomic analyses identified significant enrichment in BMI1 target genes in Osi-R cells, validating BMI1 as master regulator of transcriptional reprogramming that supports both aggressiveness and therapy escape. Notably, BMI1 downregulation reduced migratory capacity in Osi-R cells, whereas BMI1 overexpression in parental cells increased mitotic entry and induced metaphase-specific defects, particularly centrosome amplification, partially phenocopying the resistant state. These findings extend prior reports implicating BMI1 in stemness, EMT, and drug tolerance across diverse tumor types [7–9] to relapsing T790M/L858R EGFR- NSCLC.

Mechanistically, BMI1-driven plasticity promotes cell-cycle progression while simultaneously lowering the threshold for mitotic collapse, creating a state of heightened mitotic fragility. Unesbulin capitalizes on this vulnerability through its dual activity on BMI1 signaling and microtubule dynamics, converting resistance-associated mitotic dependency into a therapeutically actionable weakness. In this context, therapeutic selectivity does not rely on absolute specificity of mitotic inhibitors, but rather on the reduced tolerance of resistant cells to additional perturbations of microtubule integrity. While normal and parental cells retain sufficient mitotic robustness, resistant cells are preferentially driven toward catastrophic mitotic failure and apoptosis, defining a clear functional therapeutic window.

Together, our findings establish BMI1 as both a mediator of therapeutic escape and a driver of aggressive behavior, while identifying BMI1-associated mitotic dependency as a therapeutic vulnerability within defined EGFR-mutant backgrounds. They support the clinical evaluation of BMI1 inhibitors, alone or in combination with Osimertinib, to delay, prevent, or overcome resistance by targeting the mitotic programs that sustain refractory subclones. Such strategies have the potential to extend TKI response durability and improve patient outcomes in selected molecular contexts.

## Materials and methods

### Cell culture conditions

The parental human lung cancer cell line H1975, which harbors the EGFR L858R/T790M mutation, was obtained from ATCC. The Osimertinib-resistant variant (Osi-R) was generated by gradually exposing H1975 parental cells (Par) to increasing concentrations of Osimertinib, up to a final concentration of 1 μM, at which the cells remained viable. Cell viability was assessed using the Trypan Blue exclusion assay, and IC50 values were calculated based on the number of viable cells relative to DMSO-treated controls after 72 h of Osimertinib exposure, using an automated cell counter (Countess, Life Technologies). Data are presented as the mean of three independent experiments.

Cells were maintained in RPMI-1640 medium supplemented with 10% fetal bovine serum (FBS), 1% L-glutamine, and 1% penicillin/streptomycin, at 37 °C in a humidified incubator with 5% CO_2_. These cells were authenticated via DNA fingerprinting [25] and tested negative for mycoplasma.

### Generation of H1975 pCMV3-BMI1up and pCMV3 cells

H1975 cells were transfected with pCMV3 backbone or with a construct containing BMI1 cDNA using Fugene HD (Promega) according to the manufacturer’s protocol. 48 h after transfection, cells were placed into medium containing hygromycin B at 200 µg/ml with fresh medium replacement every 4 days. After 2 weeks, surviving cells were observed and single cells resistant to hygromycin B were transferred into 96-well plates to ensure that they could grow into hygromycin B resistant colonies. BMI1 expression was validated by determining protein and mRNA levels.

### Gene knock down assay

Cells were grown to 50-60% confluence and transfected with 20 nM siRNAs using Lipofectamine RNAiMax (#13778, Invitrogen) for 72 h. For negative controls, cells were transfected with scrambled siRNAs. The targeting sequence for Bmi1 was 5′-GAC CAG ACC ACU ACU GAA T-3′, and scrambled control sequence was 5′-UUC UCC GAA CGU GUC ACG UTT-3′.

### Enrichment analysis

The list of significantly upregulated genes was analyzed using the EnrichR platform for functional enrichment [26–28]. Enrichment analysis was performed using the MSigDB_Hallmark database, to identify overrepresented biological pathways and cellular functions. GSEA was performed on the list of genes ranked by the product between absolute log2 fold change (Osi-R vs. Par) and adjusted p-value [29]. corrected through the Benjamini-Hockberg procedure [30]. using R software environment.

### Cell collection and protein extraction

H1975 pCMV3-BMI1up and pCMV3 cells were detached from culture plates using trypsin-EDTA, centrifuged at 261x g for 5 min at room temperature, and washed with PBS. The resulting cell pellet was snap-frozen in liquid nitrogen and stored at -80 °C until further processing. To extract proteins, the cell pellet was lysed for 30 min in 1X Triton X-100 (Thermo Scientific, #A16046) supplemented with cOmplete EDTA-free Protease Inhibitor Cocktail and PhosStop Phosphatase Inhibitor Cocktail. The lysates were incubated on ice for 30 min, with brief vortexing every 10 min. After incubation, the samples were centrifuged at 12,000 × g for 20 min at 4 °C, and the supernatant was collected and stored at -80 °C.

### Immunofluorescence staining

H1975 Par and H1975 Osi-R cells were seeded on special glass slides suitable for in vitro culture and allowed to adhere for 24 h until they reached approximately 60-80% confluence. Cells were fixed with 2% formaldehyde (Sigma-Aldrich #252549) in PBS for 10 min at room temperature and washed three times with PBS for 5 min each. Then, cells were permeabilized with 0.25% Triton X-100 (Thermo Fisher Scientific #A16046) in PBS for 5 min, followed by blocking with 3% BSA (Sigma-Aldrich #A3912) and 0.2% Triton X-100 in PBS for 30 min at room temperature in a humidified chamber. After that, cells were incubated overnight at 4 °C with the appropriate primary antibodies diluted in antibody dilution buffer (1× PBS, 1% BSA, 0.3% Triton X-100). Then, cells were washed three times with PBS for 5 min each and incubated with the secondary antibodies diluted in antibody dilution buffer for 1-2 h at room temperature in the dark, and samples were washed three times with PBS for 5 min each. Nuclei were counterstained with DAPI (1 µg/mL) for 5 min at room temperature, washed three times with PBS, and samples were mounted. Images were acquired using a fluorescence microscope (Leica DM2500).

**Table:** 

Antibodies and dilution	
**Primary antibody**	**Dilution**
anti-α Tubulin mouse antibody (Abcam #7291)	1/1000 (in antibody dilution buffer 1XPBS, 1% BSA, 0.3% Triton X-100)
anti-γ Tubulin rabbit antibody (Abcam #179503)	1/500 (in antibody dilution buffer 1XPBS, 1% BSA, 0.3% Triton X-100)
**Secondary antibody**	**Dilution**
Anti-mouse Alexa 488 (Thermo Fisher Scientific #A11001)	1/1000 (in antibody dilution buffer 1XPBS, 1% BSA, 0.3% Triton X-100)
Anti-rabbit Alexa 567 (Thermo Fisher Scientific #A11011)	1/500 (in antibody dilution buffer 1XPBS, 1% BSA, 0.3% Triton X-100)
Anti-mouse Alexa Fluor 568 (Thermo Fisher Scientific #A11004)	1/500 (in antibody dilution buffer 1XPBS, 1% BSA, 0.3% Triton X-100)
Anti-rabbit Alexa Fluor 647 (Abcam #150079)	1/500 (in antibody dilution buffer 1XPBS, 1% BSA, 0.3% Triton X-100)

### Cell collection and protein extraction

H1975 pCMV3-Bmi1up and pCMV3 cells were detached from culture plates using trypsin-EDTA, centrifuged at 261x g for 5 min at room temperature, and washed with PBS. The resulting cell pellet was snap-frozen in liquid nitrogen and stored at −80 °C until further processing. To extract proteins, the cell pellet was lysed for 30 min in 1X Triton X-100 (Thermo Scientific, #A16046) supplemented with cOmplete EDTA-free Protease Inhibitor Cocktail and PhosStop Phosphatase Inhibitor Cocktail. The lysates were incubated on ice for 30 min, with brief vortexing every 10 min. After incubation, the samples were centrifuged at 12,000 × g for 20 min at 4 °C, and the supernatant was collected and stored at - 80 °C.

### SDS-PAGE and Western Blotting

For protein analysis, 15 μg of protein from each sample (H1975 Par, Osi-R, pCMV3-Bmi1up and pCMV3 cells) was separated by SDS-PAGE on 10% gel and subsequently transferred to nitrocellulose membranes with the TransBlot Transfer System (Bio-Rad). Membranes were blocked for 1 h at room temperature in 5% non-fat dry milk diluted in Tris-buffered saline with 0.1% Tween-20 (1X TBS-T). Primary antibodies (listed below) were incubated overnight at 4 °C. After incubation, membranes were washed three times for 10 min each with 1X TBS-T at room temperature. HRP-conjugated secondary antibodies (listed below) were then applied for 1 h at room temperature. Protein bands were detected by chemiluminescence (Pierce ECL Western Blotting Substrate Cat #32106) and visualized using the Chemidoc Imaging System (Biorad). Membranes were subsequently stripped using Stripping Buffer Solution (Himedia, #ML163), as per the manufacturer’s instructions, and re-probed with an anti-β-actin mouse antibody to verify equal protein loading. After three washes with 1X TBS-T, membranes were incubated with the appropriate anti-mouse secondary antibody (details below).

**Table:** 

Antibodies and dilution	
**Primary antibody**	**Dilution**
anti-β-Actin-HRP antibody (Santa Cruz #47778)	1/2000 (in 5% non-fat milk)
anti-β-Actin mouse antibody (Santa Cruz # C3022)	1/1000 (in 5% non-fat milk)
anti-β-Tubulin mouse antibody (Sigma-Aldrich #T5293)	1/1000 (in 5% non-fat milk)
anti-BMI1 rabbit antibody (Cell Signaling Technologies #6964)	1/1000 (in 5% non-fat milk)
**Secondary antibody**	**Dilution**
anti rabbit (Santa Cruz #2004)	1/5000 (in 5% non-fat milk)
anti-mouse (Immunoreagents #NC27606)	1/2000 (in 5% non-fat milk)
anti-rabbit (Cell Signaling Technologies #7074)	1/2000 (in 5% non-fat milk)

### BrDU cell proliferation assay

Cell proliferation was assessed using the BrdU Cell Proliferation Assay Kit (Sigma Aldrich, Cat. #QIA58) according to the manufacturer’s instructions. Briefly, H1975 Par and H1975 Osi-R, Bmi1-up and pCMV3 cells were seeded in 96-well plates and allowed to adhere for 24 h until they reached approximately 40–60% confluence. BrdU was added and incubated for 2–24 h at 37 °C in a humidified incubator with 5% CO_2_, after which cells were fixed. Incorporated BrdU was detected with mouse anti-BrdU antibody (1/1000), followed by HRP-conjugated anti-mouse IgG secondary antibody (1/1000). Substrate solution was added and incubated for 15 min at room temperature and then the reaction was stopped with the stop solution. Absorbance was measured at 450 nm using a spectrophotometric plate reader.

### Transwell migration assays

Transwell migration assays were performed using Boyden Chamber from Corning® BioCoat™ Matrigel® Invasion Chambers (cat #354480) with 8.0 µm PET Membrane in two 24-well plates. Boyden chamber membranes were rehydrated by adding 400 μL of 1% FCS media to the upper chamber and 700 μL of 1% FCS media to the lower chamber, followed by incubation for 2 h at 37 °C in a humidified incubator with 5% CO₂. H1975 Par, Osi-R, Bmi1-up and pCMV3 cells were seeded onto the upper chamber, while 700 μL of complete culture medium supplemented with 10% FCS was added to the lower chamber as a chemoattractant. Cells were allowed to migrate for 48 h under standard culture conditions (37 °C, 5% CO₂). Then, cells were fixed with 1% paraformaldehyde for 10 min at room temperature, washed twice with PBS, and permeabilized with 0.2% Triton X-100 for 10 min. Cells that migrated to the lower surface of the membrane were stained with DAPI (1:1000) for 10 min. Images were taken with a fluorescence microscope at 5x and 10 x magnification.

### Wound healing assays

H1975 Par and Osi-R cells were seeded in 6 well plates and cultured until 90-100% confluence was reached. A linear scratch was made across the center of each well using the tip of a sterile 200 μL pipette, ensuring comparable wound widths across replicates. Then, the 10% FBS growth medium was replaced with culture medium supplemented with 2% FBS to minimize cell proliferation during the assay. Images of the wound area were acquired at 0 h, 8 h, 12 h, 14 h, and 16 h. Image processing was performed using the “Wound healing size tool” plugin in ImageJ/Fiji® software (Version 1.53c, NIH, Bethesda, MD).

### PTC596/Unesbulin treatment

H1975 Osi-R cells were treated with Unesbulin (1 μmol/L), or Vehicle (0.5% DMSO) for various time points (3, 12, 24, 48, and 72 h). Cells were harvested, proteins extracted, and Western Blot was performed as detailed above.

Apoptosis was assessed using the Annexin V Apoptosis Detection Kit FITC (Invitrogen, Cat. #88-88005-74) following the manufacturer’s instructions. Briefly, cells were harvested, filtered through cell strainer caps into FACS tubes (Falcon, Cat. #352235) and resuspended in Annexin V binding buffer 1X. Annexin V working solution (1/20 dilution) was added to each sample and incubated for 10 min in the dark. Cells were washed with binding buffer, centrifuged at 1200 rpm for 5 min at room temperature, and the supernatant was discarded. Propidium iodide (PI) working solution (1/40 dilution) was added. Samples were analyzed within 4 h by flow cytometry. For each time point the following controls were included:

- A positive control only stained with Annexin V

- A positive control only stained with PI

- A positive control stained with both AnnexinV and PI

- A negative control without AnnexinV and PI staining

FlowJo version 10.0 was used to analyze the apoptosis data.

### Xenograft studies and drug treatments

B-NDG (NOD.CB17-Prkdcscid IL2rgtm1/Bcgen, Envigo) mice were used for xenograft studies. For subcutaneous tumor establishment, 1×10⁶ H1975 Par, Osi-R, Bmi1-up or pCMV3 cells were resuspended in 50 μL of Matrigel Basement Membrane Matrix, Phenol Red-free (BD Biosciences, #356237) and injected into both flanks of mice.

Tumor growth was monitored weekly by high-resolution ultrasound imaging beginning on day 10 post-implantation and followed for 3 weeks. To evaluate the growth of the cells in vivo, tumor volume (mm³) was calculated from ultrasound measurements, and growth kinetics were compared. Maximal tumor size/burden was not exceeded.

To study the effect of Unesbulin in vivo, H1975 Osi-R xenografts were randomized to receive Unesbulin (12 mg/kg), or Vehicle (0.5% hydroxypropyl methyl cellulose-HPMC-and 0.2% Tween 80 in distilled water) by oral gavage twice a week, once the tumor became measurable.

For Osimertinib response studies, xenografts with H1975 Osi-R, H1975 Bmi1-up and H1975 pCMV3 cells received biweekly treatment with Osimertinib (10 mg/kg, oral gavage) or vehicle control, once tumors became measurable. Mice were housed in a sterile-barrier facility, and all experiments were approved by the Italian Ministry of Health (627/2021-PR, 488/2022-PR, and 581/2024-PR). All methods were performed in accordance with the relevant guidelines and regulations.

### High resolution ultrasound imaging

Ultrasound (US) imaging was performed using the multimodal imaging platform Vevo LAZR-X system (FUJIFILM VisualSonics Inc., Toronto, Canada) using high-resolution ultrasound probes. The system is equipped with a sensorized animal bed for physiological monitoring and temperature control. Mice were initially anesthetized in an induction chamber with 2% isoflurane and then moved to the sensorized bed, where anesthesia was maintained via a nose cone with 1% isoflurane. The bed temperature was set around 40 °C throughout the procedure to prevent hypothermia. Depending on the required field of view, two transducers were employed: the X550D probe (frequency range: 25–55 MHz; central band: 40 MHz, axial resolution 40 µm), and the MX250 probe (frequency range: 15–30 MHz; central band: 25 MHz, axial resolution 75 µm).

Tumor morphology was assessed by three-dimensional (3D) imaging of the region of interest using a motorized stepper system (step size: 150 µm), allowing for high-resolution volumetric reconstruction of the tumor area. Coupling between the US probe and tumors was ensured by US clear gel (Cogel, Comedical s.r.l.), ensuring optimal acoustic transmission.

Quantification was performed using Vevo Lab ultrasound analysis software.

### Statistics

Statistical analyses were performed using Graphpad Prism 10.4.1 (532). Specific details are provided within each figure legend.

## Supplementary information

Original Data

Supplementary Figures

## Data Availability

RNA sequencing data were deposited into EMBL-EBI’s ArrayExpress under accession number E-MTAB-15298.
