# Supplementary material for: Dual actionability of BMI1 activation and mitotic vulnerability defines adaptive Osimertinib resistance in EGFR-mutant NSCLC
Source: Cell Death Dis. 2026 Jun 10;17(1):732. doi: 10.1038/s41419-026-08952-2 (PMC13478582; doi:10.1038/s41419-026-08952-2)
Supplement: Supplementary file 1 [file 41419_2026_8952_MOESM2_ESM.docx]

**Supplementary Figure 1.** A, Viability assay for Par (blue) and Osi-R cells (red) after 72h exposure to increasing Osimertinib concentrations. Dashed grey line indicates 50% viability. IC50 for Par cells is ~0.5 μM and for Osi-R cells is ~3 μM. Black dotted rectangle highlights 80% of viable Osi-R cells at 1 μM of Osimertinib. n=3. Error bars represent SD; p-value are indicated (***p<0.0002, unpaired t test with Welch's correction, no correction for multiple comparisons).

B, Wound-healing assay images of Par (top panel) and Osi-R (bottom panel) H1975 cells at the indicated time points. (c) Quantitative analysis of wound healing assay of H1975 Par (blue) and Osi-R (red) cells. n=3. Error bars represent SD; p-value are indicated (*p<0.032, **p<0.0021, ***p<0.0002, Multiple Mann-Whitney Tests).

**Supplementary Figure 2. BMI1 down regulation decreases migration capacity in Osi-R cells**

A, Western Blot analyses of H1975 Osi-R cells treated with scramble or Bmi1 siRNAs (20nM). Protein lysates were immunoblotted with an anti-BMI antibody. Expected molecular weights (kDa) are indicated. B, Representative transwell migration images of DAPI-stained nuclei in H1975 Osi-R treated with scramble siRNA (left panel) and Bmi1 siRNA (right panel). C, Quantification of transwell migration data; Osi-R cells treated with scramble siRNA are shown as black bar, and Bmi1 siRNA-treated cells in red. Error bars represent SD; p-value is indicated (***p<0.001, unpaired T Test with Welch’s correction, n=3).
